# Clinical features, outcome and risk factors in cervical cancer patients after surgery for chronic radiation enteropathy

**DOI:** 10.1186/s13014-015-0433-5

**Published:** 2015-06-06

**Authors:** Jianbo Yang, Chao Ding, Tenghui Zhang, Liang Zhang, Tengfei Lv, Xiaolong Ge, Jianfeng Gong, Weiming Zhu, Ning Li, Jieshou Li

**Affiliations:** Research Institute of General Surgery, Jinling Hospital, Medical School of Nanjing University, No. 305 East Zhongshan Road, Nanjing, 210002 People’s Republic of China; Research Institute of General Surgery, Jinling Hospital, Clinical School of Nanjing, Southern Medical University, No. 305 East Zhongshan Road, Nanjing, 210002 People’s Republic of China

**Keywords:** Cervical cancer, Chronic radiation enteropathy, Surgery, Radical radiotherapy, Radical surgery

## Abstract

**Background:**

Radical hysterectomy and radiotherapy have long been mainstays of cervical cancer treatment. Early stage cervical cancer (FIGO stage IB1–IIA) is traditionally treated using radical surgery combined with radiotherapy, while locally advanced cervical cancer is treated using radiotherapy alone or chemoradiotherapy. In this retrospective study, we describe and analyse the presenting clinical features and outcomes in our cohort and evaluate possible risk factors for postoperative morbidity in women who underwent surgery for chronic radiation enteropathy (CRE).

**Methods:**

One hundred sixty-six eligible cervical cancer patients who underwent surgery for CRE were retrospectively identified between September 2003 and July 2014 in a prospectively maintained database. Among them, 46 patients received radical radiotherapy (RRT) and 120 received radical surgery plus radiotherapy (RS + RT). Clinical features, postoperative morbidity and mortality, and risk factors for postoperative morbidity were analysed.

**Results:**

RS + RT group patients were more likely to present with RTOG/EORTC grade III late morbidity (76.1 % vs 92.5 %; *p* = 0.004), while RRT group patients tended to show RTOG/EORTC grade IV late morbidity (23.9 % vs 7.5 %; *p* = 0.004). One hundred forty patients (84.3 %) were treated with aggressive resection (anastomosis 57.8 % and stoma 26.5 %). Overall and major morbidity, mortality and incidence of reoperation in the RRT and RS + RT groups did not differ significantly (63 % vs 64.2 % [*p* = 1.000], 21.7 % vs 11.7 % [*p* = 0.137], 6.5 % vs 0.8 % [*p* = 0.065] and 6.5 % vs 3.3 % [*p* = 0.360], respectively). However, incidence of permanent stoma and mortality during follow-up was higher in the RRT group than in the RS + RT group (44.2 % vs 12.6 % [*p* = 0.000] and 16.3 % vs 3.4 % [*p* = 0.004], respectively). In multivariate analysis, preoperative anaemia was significantly associated with overall morbidity (*p* = 0.015), while severe intra-abdominal adhesion (*p* = 0.017), ASA grades III–V (*P* = 0.022), and RTOG grade IV morbidity (*P* = 0.018) were predicators of major morbidity.

**Conclusions:**

Radiation-induced late morbidity tended to be severe in the RRT group with more patients suffering RTOG/EORTC grade IV morbidity, while there were no significant differences in postoperative morbidity, mortality and reoperation. Aggressive resection was feasible with acceptable postoperative outcomes. Severe intra-abdominal adhesion, ASA grades III–V and RTOG/EORTC grade IV late morbidity contributed significantly to major postoperative morbidity.

## Background

On a global scale cervical cancer is the fourth most common cancer in women and the seventh overall, with an estimated 528,000 new cases and 26,000 deaths each year [1]. Radical hysterectomy and radiotherapy (RT) have long been mainstays of treatment for cervical cancer. Traditionally, early stage cervical cancer (FIGO stage IB1–IIA) has been treated with either primary surgery, with or without combined RT, or with definitive RT [2–6]. FIGO stage IIB-IVA is recognized as a locally advanced cervical cancer (LACC) and consequentially RT alone or chemoradiotherapy has been considered as the standard treatment [7, 8]. According to the National Comprehensive Cancer Network (NCCN) guidelines version 2.2015, FIGO stage IB2 and IIA2 are included in the advanced disease category; cisplatin-based chemoradiotherapy is recommended as the primary treatment [8].

Despite the improvement in techniques and the availability of better equipment, chronic radiation enteropathy (CRE) has remained a major problem in patients undergoing pelvic RT [9]. The reported incidence of late gastrointestinal toxicities from RT varies from 8 % to 50 % in cervical cancer patients [10–12, 3, 13, 14]; some of the patients with CRE will require surgery to treat obstruction, fistulas, perforation or bleeding [15].

With progress in perioperative management and surgical techniques, definitive surgery has gained popularity as the first choice for patients with CRE [16]. Iraha *et al.* [17] retrospectively reviewed 1349 patients with gynaecological malignancies who had received radiotherapy (1132 patients with cervical cancer), and reported that liberal resection of the affected bowel appears to be the preferable therapy in patients with radiation enterocolitis. Furthermore, severe radiation enterocolitis requiring surgery usually occurred at the terminal ileum and is strongly correlated with previous abdominopelvic surgery, diabetes mellitus, and smoking [17]. However, there has been limited information reported, especially regarding the clinical features and outcome in cervical cancer patients treated for surgery for CRE in a large cohort study.

The aim of current study was twofold: to describe the clinical features, postoperative morbidity in cervical cancer patients who underwent surgery for CRE; and to identify possible risk factors for postoperative complications.

## Methods

### Patients

We retrospectively reviewed the medical records of 196 patients with CRE after pelvic RT for cervical cancer, from September 2003 to July 2014, in a prospectively maintained database at a tertiary-care referral institution. Patients treated with previous palliative RT (*n* = 4), and with tumour recurrence (*n* = 10) or a pelvic neoplasm (*n* = 1), or who underwent nonsurgical treatment (*n* = 15) were excluded from the present study. The diagnosis of CRE was confirmed by intraoperative findings and postoperative pathology. Finally, 166 eligible patients requiring surgery for CRE were included in the study; 46 patients had previously received radical radiotherapy (RRT) without gynaecological surgery, and 120 patients had received radical surgery plus RT (RS + RT) for cervical cancer. The cohort study was approved by the ethics committee of Jinling Hospital.

Data on the following parameters were extracted from the patients’ medical records: demographics; FIGO tumour stage; cumulative dose; latency period between the first symptoms and completion of RT; time interval from disease onset to surgery; nutritional status; clinical manifestations; perioperative parameters; postoperative morbidity; and mortality. Perioperative parameters including preoperative total parenteral nutrition (TPN) dependence, American Society of Anesthesiologists (ASA) grade, surgical procedures, operation time and intraperitoneal adhesion states.

### Grading criteria

The grading system used for late radiation gastrointestinal morbidity was in accordance with the criteria of the Radiation Therapy Oncology Group (RTOG) and the European Organization for Research and Treatment of Cancer (EORTC), with grade III for obstruction or bleeding requiring surgery, and grade IV for necrosis/perforation or fistula [18].

The severity of intraperitoneal adhesion was assessed by the operating surgeons and graded from I to V according to the scale that Hobson *et al.* reported [19]. Patients with no adhesion were defined as grade I. Patients with moderate adhesion included grade II (minimal adhesions localised to one or two areas) and grade III (diffuse adhesions, but not extensive). Patients with severe adhesion included grade IV (diffuse, extensive adhesions easily lysed) and grade V (diffuse, extensive, dense adhesions difficult to lyse).

Postoperative morbidity was graded according to the Clavien–Dindo classification [20]. Postoperative TPN dependence for >2 weeks was considered as grade II morbidity. For patients who underwent staged procedures (*i.e.*, stoma in the first operation and stoma reversal in the second operation), the complication rate was calculated as the total events encountered in two operations.

### Follow-up

All patients were followed up until death or July 2014, when the data were collected. The long-term outcomes, including TPN dependence, tumour recurrence and reoperation for CRE were evaluated. In addition, patient survival status and the presence of a definite stoma at the end of follow-up were also recorded by review of the medical records, and by telephone follow-up of all patients who had no regular postoperative outpatient visits for >3 months.

### Statistical analysis

Results are presented as the mean ± SD or median (range) for continuous variables, except for categorical variables which are presented as numbers. Statistical analysis was performed using Student’s *t* test for continuous variables and Pearson’s chi-square test or Fisher’s exact test for categorical variables as appropriate. Potential risk factors for postoperative morbidity were evaluated using univariate analysis, and risk factors with p < 0.10 were included in the multivariate analysis using multivariate logistic regression analysis. Survival status was analysed using the Kaplan–Meier method and distributions were compared using the log rank test. Result with p < 0.05 were considered significant. All analyses were performed using the Statistical Package for Social Sciences software (version 19.0; SPSS, Inc., Chicago, IL, USA).

## Results

### Patient characteristics

Patients in the RRT group mostly had LACC, with 92.9 % of patients staged as IIb–Va; patients in the RS + RT group had early stage cervical cancer, with 86.7 % of patients staged as I–IIa. Patients in the RRT group were older than those in the RS + RT group (51.8 years vs 47.8 years; p < 0.05). The total radiation dose (external beam plus brachytherapy) was known for 107 (64.5 %) patients, with a mean dose of 72.6 Gy and 50.4 Gy in the RRT and RS + RT groups, respectively. The median latency period from completion of radiotherapy to disease onset in the RS + RT group tended to be shorter than in the RRT group (6 months vs 9 months; *p* = 0.000). The baseline demographics are presented in Table 1.

**Table 1:** Demographic data of 166 cervical cancer patients requiring surgery for chronic radiation enteropathy

Characteristics	RRT (*n* = 46)	RS + RT (*n* = 120)	*P* value
Age, y (mean ± SD)	51.8 ± 10.4	47.8 ± 9.4	0.020
Tumor stage, n (%)^a^			0.000
I-IIa	3(7.1)	78(86.7)	
IIB-IVa	39(92.9)	12(13.3)	
Cumulative dosage, Gy (mean ± SD)^b^	72.6 ± 22.0	50.4 ± 13.1	0.000
Preoperative, n (%)	-	5 (4.17)	
Postoperative, n (%)	-	111 (92.5)	
Pre/postoperative, n (%)	-	4 (3.33)	
Chemotherapy, n (%)	26(56.5)	57(47.5)	0.298
Pathological pattern, n (%)^c^			0.147
squamous carcinoma	40 (90.9)	94 (79.7)	
adenocarcinoma	4 (9.1)	14 (20.3)	
Acute radiation enteritis, n (%)^d^	16/38 (42.1 %)	42/112(37.5 %)	0.614
Median latency period, n (%) Median (range), n (%)	9(3–264)	6(3–129)	0.000
≦6	13 (28.3)	70 (58.3)	0.001
7–12	17 (37.0)	33 (27.5)	0.235
13–24	6 (13.0)	7 (5.8)	0.122
>24	10 (21.7)	10 (8.3)	0.018
Time interval from disease onset to referral Median (range)	6.5(1–108)	5.5(0.5–152)	0.295
BMI, mean ± SD (range) N (%)	19.0 ± 3.1	17.8 ± 3.2	0.025
<=18.5	23(50.0)	75(65.8)	0.074
>18.5	23(50.0)	39(34.2)	0.074
hypertension	3 (6.5)	9 (7.5)	1.000
Diabetes mellitus	2(4.3)	2(1.7)	0.658
Double j-tube placement, n (%)	4 (8.7)	1 (0.8)	0.021
Blood infusion, n (%)	9 (19.6)	29 (24.2)	0.528
Previous abdominal surgery, n (%)	21(45.7)	36(30.0)	0.086

*RRT* Radical Radiotherapy, *RS + RT* Radical Surgery plus Radiotherapy, *SD* standard deviation, *BMI* body mass index

^a^FIGO tumor stage was unknown in 34 patients (4/30)

^b^Radiation dose was unknown in 39 patients (11/28)

^c^Pathological pattern was unknown in 14 patients (2/12)

^d^Acute radiation enteritis was unknown in 16 patients (8/8)

### Clinical manifestations

The main clinical manifestation in the present study was clustered into four broad categories: stenosis/obstruction (139/166); fistula (15/166); free perforation (5/166); and severe chronic radiation proctitis (SCRP) (7/166). The main symptoms of CRE on admission are presented in Table 2. Considered overall, patients in the RS + RT group were more likely to present with RTOG/EORTC grade III late morbidity (obstruction/SCRP) (92.5 % vs 76.1 %; *p* = 0.004), while patients in the RRT group tended to show RTOG/EORTC grade IV late morbidity (fistula/perforation) (23.9 % vs 7.5 %; *p* = 0.004). Intestinal or colorectal stricture responsible for complete or incomplete obstruction was the most common symptom with 65.2 % in the RRT group and 90.8 % in the RS + RT group (*p* = 0.000). Fistula, the second most common symptom, was inclined to appear more often in the RRT group than in the RS + RT group (17.4 % vs 5.8 %; *p* = 0.020). Although not statistically significant, the incidence of perforation in the RRT group was five times higher than that in the RS + RT group (6.5 % vs 1.7 %; *p* = 0.258). Fourteen (30.4 %) patients in the RRT group and 10 (8.3 %) in the RS + RT group suffered from chronic radiation proctitis, and chronic radiation proctitis was the main reason for surgery in seven of them.

**Table 2:** Clinical manifestation and surgical parameters of 166 patients requiring surgery for chronic radiation enteropathy

Characteristics	RRT (*n* = 46)	RS + RT (*n* = 120)	*P* value
Surgical procedure, n (%)			
Obstruction	30(65.2)	109(90.8)	0.000
Ileal R/A	9(15.2)	41(34.2)	
Ileocecal R/A	7(23.3)	39(32.5)	
Ileal/Ileocecal R+ ileostomy	8(17.4)	22(18.3)	
Ileal/Ileocecal R/A+ colostomy	2(4.3)	1(0.8)	
Ileostomy	3(6.5)	2(1.7)	
Colostomy	-(0)	1(0.8)	
Intestinal enterolysis + Intestinal	1(2.2)	0(0)	
Intubafion Plicafion			
Fistula	8(17.4)	7(5.8)	0.020
Ileal R+ ileostomy	1(2.2)	-	
Ileal R/A+ colostomy	1(2.2)	-	
Ileocecal R/A+ colostomy	1(2.2)	3(2.5)	
Ileal R+ ileostomy + colostomy	1(2.2)	-	
Ileal R/A+ neobladder + colostomy	1(2.2)	-	
Colostomy	3(6.5)	4(3.3)	
Free perforation	3(6.5)	2(1.7)	0.258
ileostomy	2(4.3)	2(1.7)	
Ileocecal R/A+ colostomy	1(2.2)	-	
Severe chronic radiation proctitis^a^	5(10.9)	2(1.7)	0.027
Colostomy	4(8.7)	1(0.8)	
Ileocecal R/A+ colostomy	1(2.2)	1(0.8)	
Total diversion stomy, n (%)	26(56.5)	36(29.8)	0.001
Ileostomy	16(34.8)	24(20.0)	0.067
Colostomy	15 (32.6)	11 (9.1)	0.000
Intraperitoneal adhesion states, n (%)^b^			
None(I)	12 (41.4)	8 (8.9)	0.000
Moderate(II-III)	12(41.4)	65(72.2)	0.003
Severe(IV-V)	5(17.2)	17(18.9)	0.842

*RRT* Radical Radiotherapy, *RS + RT* Radical Surgery plus Radiotherapy, *R/A* Resection/Anastomosis, *R* resection

^a^Severe chronic radiation proctitis as the main clinical manifestation requiring surgery

^b^Intra-peritoneal adhesion states in patients without suffer other abdominal/pelvic surgery before transferred to our center, except for previous pelvic surgery for cervical cancer

### Surgical procedures

Details of the surgical parameters are shown in Table 2. In the study, 143 patients underwent aggressive resection procedures; 13 (28.3 %) patients in the RRT group and 10 (8.4 %) patients in RS + RT group merely underwent ileostomy or colostomy because of poor nutritional status, severe intraperitoneal adhesion or radiation-induced recto-sigmoid lesions. The state of intraperitoneal adhesion was much more severe in the RS + RT group than in the RRT group in patients who did not receive additional abdominal/pelvic surgery except for primary surgery for cervical cancer. However, radiation-induced gastrointestinal lesions were extensive in the RRT group with more patients undergoing colostomy for severe recto-sigmoid disease (32.6 % vs 9.1 %; *p* = 0.000).

### Postoperative morbidity

Sixty (36.1 %) patients experienced no adverse events and recovered uneventfully; however, 29 (63.0 %) in the RRT group and 77 (64.2 %) in the RS + RT group experienced postoperative morbidity. Major (grades III–V) morbidity was higher in the RRT group than in the RS + RT group, but did not differ significantly (21.7 % vs 11.7 %; *p* = 0.137). Postoperative morbidity according to the Clavien–Dindo classification is detailed in Table 3. Four patients died during the postoperative course; one patient died of uncontrolled intra-abdominal haemorrhage, and the other three from intra-abdominal sepsis and multiple organ dysfunction syndrome (MODS). Surgical complications occurred in 16.3 % of patients (8 in the RRT group vs 19 in the RS + RT group); among these patients, three in the RRT group and four in RS + RT group required relaparotomy.

**Table 3:** Postoperative complications according to Clavien-Dino classification in 166 cervical cancer patients after surgery for chronic radiation enteropathy

Postoperative complication	RRT (*n* = 46)	RS + RT (*n* = 120)	*P* value
Grade I	5(10.9 %)	21(17.5 %)	0.348
Diarrhea	3	11	
Incisional infection	2	9	
Delayed gastric emptying	-	1	
Grade II	14(30.4 %)	42(35.0 %)	0.590
Total Parenteral Nutrition(TPN) > 2w	4	8	
TPN > 2w/Blood transfusion	2	7	
Blood transfusion	6	17	
Catheter infection	1	7	
Urinary infection	1	1	
Early postoperative obstruction	-	2	
Grade III	5(10.9 %)	12(10.0 %)	1.000
Pleural effusion and drainage	1	4	
Seroperitoneum and drainage	1	2	
Anastomotic leakage	1	-	
Gastrointestinal hemorrhage	1		
Cholestasis and biliary drainage	-	2	
Bladder puncture and drainage	-	1	
Would infection/bleeding/dehiscence	-	1	
Intestinal fistula	1		
Incomplete resection and reoperation	-	2	
Grade IV	2(4.3 %)	1(0.8 %)	0.186
Renal failure	1	-	
Anastomosis leakage and heart failure	1	-	
Intestinal fistula and respiratory failure		1	
Grade V	3(6.5 %)	1(0.8 %)	0.065

### Risk factors for postoperative morbidity

Factors associated with overall and major postoperative morbidity in univariate and multivariate analysis are listed in Table 4. In multivariate analysis, preoperative anaemia (*p* = 0.015) was found to be a significant predictor for overall postoperative morbidity, while severe intraperitoneal adhesion at the surgical site (grades IV–V; *p* = 0.017), ASA grades III–V (*p* = 0.022), RTOG/EORTC grade IV late morbidity (*p* = 0.018) were significantly associated with major morbidity.

**Table 4:** Univariant and multivariant analysis of potential factors associated with postoperative complications

	Overall morbidity			Major morbidity		
	(grade I–V)			(grade III–V)		
	Univariant	Multivariant		Univariant	Multivariant	
Variables	*P* value	*P* value	OR(95 % CI)	*P* value	*P* value	OR(95 % CI)
Anemia (Y/N)	0.023	0.015	2.626(1.204–5.728)	0.473	-	-
Time interval from disease onset to surgery (<6 m/>6 m)	0.000	0.062	0.527(0.269–1.364)	0.181	-	-
Chemotherapy (Y/N)	0.023	0.307	0.7(0.353–1.388)	1.000	-	-
Operation time	0.092	0.210	1.588(0.770–3.277)	0.816	-	-
(<150/>150) min						
Adhesion states in surgical site (IV-V/I-III)	0.046	0.089	2.180(0.887–5.360)	0.066	0.017	3.535(1.250–10.001)
ASA grade (III–V/I-II)	1.000	-	-	0.014	0.022	3.400(1.197–9.653)
RTOG/ETORC Grade IV late morbidity (Y/N)	0.129	-	-	0.001	0.018	0.171(0.040–0.735)
Stomy surgery (Y/N)	0.617	-	-	0.039	0.575	1.723(0.257–11.546)
Staged surgery (I/II)	0.492	-	-	0.099	0.735	0.731(0.120–4.474)
Age (<50/>50) y	0.624	-	-	1.000	-	-
Tumor stage (I-IIa/IIb-V)	0.496	-	-	0.702	-	-
Pathological type (squamous carcinoma/adenocarcinoma)	0.710	-	-	1.000	-	--
Radiation dosage (Gy)	0.373	-	-	0.379	-	-
Acute radiation enteritis (Y/N)	0.475	-	-	0.807	-	-
Previous abdominal surgery (Y/N)	0.345	-	-	0.789	-	-
Latency period (m)	0.194	-	-	0.369	-	-
Hypertention (Y/N)	0.569	-	-	0.108	-	-
Diabetes mellitus (Y/N)	0.616	-	-	0.453	-	-
BMI < 18.5 and weight loss >10 %	0.217	-	-	1.000	-	-
Preoperative TPN (Y/N)	0.407	-	-	0.493	-	-
Short bowel syndrome (Y/N)	0.330	-	-	1.000	-	-

*RTOG/ETORC* the Radiation Therapy Oncology Group (RTOG) and the European Organization for Research and Treatment of Cancer (EORTC), *ASA* American Society of Anesthesiologists, *BMI* Body mass Index

### Follow-up

**Fig. 1:**
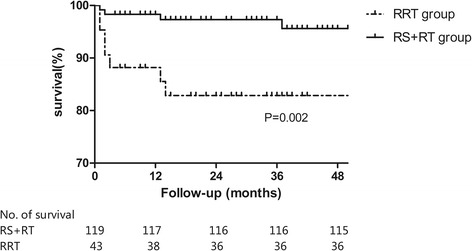
Survival states in cervical cancer patients after surgery for chronic radiation enteropathy (*n* = 162). Seven patients in RRT and four patients in RS + RT group died during follow-up period. In-hospital deaths were excluded from survival analysis

Mean and median follow-up times were 29 months and 26 (range, 5–70) months, respectively in the RRT group, and 36 months and 33 (range, 5–121) months in the RS + RT group. Seven patients in the RRT group and four in the RS + RT group died during the follow-up period (Fig. 1). Of note, seven patients died of MODS caused by intra-abdominal leakage and sepsis within 3 months after discharge, while the other patients died of tumour recurrence at 1 year after discharge. Seven patients exhibited symptoms of recurrence of CRE, and each of two groups had two patients requiring reoperation. However, only one patient could not be weaned off TPN because of short bowel syndrome. By the end of the follow-up period, a definitive stoma was present in 19 (44.2 %) patients in the RRT group as compared with 15 (12.6 %) in RS + RT group (*p* = 0.000).

## Discussion

Because the management of cervical cancer is stratified by tumour stage, we undertook this retrospective study to analyse the clinical characteristics and postoperative outcomes for inpatients with CRE requiring surgery after RRT or RS plus RT. In this series of 166 consecutive cases of CRE requiring surgery, more patients suffered from radiation-induced fistula, perforation and proctitis in the RRT group. An aggressive intestinal resection procedure was performed in almost all of the cases with overall and major morbidity rates of 63.9 % and 14.5 %, respectively. Although the surgical procedure is very difficult in patients with CRE, extensive resection of irradiated bowel is suitable and feasible with acceptable postoperative outcomes.

Radiation induced digestive stenosis, which was responsible for complete or incomplete obstruction, was the most common symptom and surgical indication (81.9 %); this finding is consistent with the previous literature [21–23]. Concerning the site of obstruction, our results indicated that the terminal ileum/ileocecum was the most frequently and severely affected site. Several studies have also reported similar results [23, 17]. Iraha *et al.* [17] reported that 37 out of 48 gynaecological cancer patients developed radiation induced stenosis requiring surgical management, and found that the terminal ileum, sigmoid and rectum were the sites of the majority of the radiation-induced lesions. The current case series revealed that the incidence of radiation-induced stenosis in the RS + RT group was higher than in the RRT group (90.8 vs 65.2 %; *p* = 0.000). A possible explanation for the increased incidence of obstruction could be the gynaecological surgical procedure for cervical cancer, during which surgical extirpation of the uterus or other organs allowed normal bowel fall within the pelvic radiation field [17]. In addition, postoperative adhesion can affect the normal mobilization of the intestine, which causes the small bowel loops to be fixed in the pelvis. The fact that the intestine is more sensitive to radiotherapy and the presence of an impaired vascular supply resulting from the surgical procedure might have aggravated the damage to the irradiated intestinal.

Fistula and SCRP are probably the most severe and feared late toxicities following RT. As a result of symptoms including leaking urine or stools, persistent bleeding, vulnerability to infection and unbearable pain, patients may suffer significant physical, social and psychological distress which negatively impacts on their quality of life [24, 25]. The incidence of radiation-induced fistulas was estimated as between 1 % and 4 % for all-comers, while this number can be as high as 22–48 % for more advanced stages, which is similar to our findings [24, 26]. In the current series, the incidence of fistula and SCRP (as the main symptoms of patients who underwent surgery) in the RRT group was significantly higher than that in the RS + RT group (17.4 % vs 5.8 % [*p* = 0.020] and 10.9 % vs 1.7 % [*p* = 0.027], respectively). The probable reason was that most patients in the RRT group had advanced stage disease and received a higher cumulative radiation dose, which correlated closely with an increased incidence of late toxicities following RT. Correlations between the radiation dose and the incidence of sequelae have been reported by many authors [27, 28]. In a review of 1456 patients with cervical carcinoma (stages IB–IVA) treated with RT (70–90 Gy), Perez *et al.* [28] quantified the impact of various dosimetric parameters on the incidence of significant morbidity. They observed an incidence of recto-sigmoid morbidity of < 4 % with doses below 75 Gy and 9 % with higher doses; for the small intestine the incidence of morbidity was < 1 % after a total dose of ≤50 Gy, 2 % after 50–60 Gy and 5 % after higher doses. Previous series have noted a variety of risk factors for severe late toxicities, but common predictors tend to include advancing tumour stage, previous pelvic radiotherapy, the use of RS, an active smoking habit and elevated RT doses [29, 11, 26]. The poor wound-healing characteristics that increase susceptibility to fistula development can largely be attributed to sclerosis in small and medium sized blood vessels, relative tissue hypoxia and soft tissue fibrosis that occurs following RT [25].

Most patients with refractory complications had more than one radiation-induced late toxicity, which increased the complexity of the disease [30]. In the current study, 42 patients underwent both ileal/ileocecal resection and stromal diversion because of radiation-induced multiple injuries. Turina *et al.* [30] also reported that over two-thirds of their patients developed two or more complications from RT, and many patients required a major operation with faecal diversion and subsequent restorative operations as a result of the severity of the RT injuries.

Although not statistically significant, the incidence of perforation in the RRT group observed in the present series was five times higher than that in the RS + RT group (6.5 % vs 1.7 %; *p* = 0.258); this was probably caused by the limited number of patients in the present study. We observed that the perforation site was the ileum in all five cases with abdominal pain as the prominent complaint, while only one (20 %) patient showed signs of acute peritonitis on physical examination. Yamashita *et al.* [31] also reported seven cases of small bowel perforation without tumour recurrence after pelvic radiotherapy for cervical cancer, and found that signs of peritonitis were absent in six (86 %) cases with severe abdominal pain as the main complaint. The absence of signs of acute peritonitis might have been attributable to the nature of its histopathological features, including obliterative endarteritis and progressive stromal fibrosis in the submucosal/subserosal layers, which limited inflammation resulting from severe abdominal adhesion.

Optimal surgical strategies for patients with CRE remain controversial. Various surgical procedures have been proposed, including aggressive resection/anastomosis, adhesiolysis, stoma formation and bypass. According to the experience from our centre and others [16, 32, 17, 12], the optimal procedure for CRE is resection and anastomosis, and the avoidance of bypass or other conservative procedures. If there is severe dense adhesion or poor general condition, a conservative procedure could be considered as an alternative option. Aggressive resection of the radiation-induced lesions, if possible, would improve the long-term outcomes after surgery. Lefevre *et al.* [21] demonstrated the importance of resecting all damaged tissue in patients with CRE, and reported that as compared with bypass or adhesiolysis, ileocecal resection was the only factor that protected against reoperation for recurrence.

In the present study, 140 (84.6 %) patients received an aggressive resection procedure (57.8 % with anastomosis and 26.5 % with ileostomy/colostomy) with surgical complications in 16.3 % and reoperation in 4.2 % of patients.

Overall and major postoperative morbidity, mortality and the incidence of reoperation in the two groups did not differ significantly. According to the updated Clavien–Dindo classification [20], overall postoperative morbidity and major (grades III–V) morbidity were observed in 63.8 % and 14.5 % of patients, respectively. Postoperative mortality was 2.4 % and incidence of reoperation was 4.8 %, which is comparable or lower than in previous reported series [33, 21]. Lefevre *et al.* [21] analysed 107 patients after extensive resection surgery for CRE and reported that the overall morbidity, surgical morbidity and postoperative mortality were 74.8 %, 28.0 % and 0.9 %, respectively. An earlier study by Regimbeau *et al.* [33] reported that postoperative surgical complications and mortality were 29 % and 5 %, respectively; anastomotic leakage occurred in 9 % of patients after aggressive surgery for CRE. Most patients in the present study experienced a planned procedure and nutritional support before surgery; this might have partly contributed to the decreased postoperative morbidity. In addition, nearly two-thirds of patients were operated in recent three years and given a targeted intervention following a detailed clinical algorithm, which could also have effectively improved outcomes.

During follow-up, mortality in the RRT group was significantly higher than that in the RS + RT group; seven patients died of MODS caused by intra-abdominal leakage or sepsis and four as a result of tumour recurrence. Of note, death secondary to postoperative complications usually occurred within 3 months after discharge, while patients with tumour recurrence tend to died 1 year later. Therefore, we believed that the focus should be on postoperative complications during the first 3 months after treatment, and then be transferred to tumour monitoring. In addition, the high incidence of permanent stoma in the RRT group might be demonstrated by the higher incidence of fistula and SCRP as a result of escalation of the radiation dose and advanced tumour stage. Because a fistula occurring in irradiated tissue can rarely be successfully repaired, most surgical treatments are palliative in the form of faecal or urinary diversion, leaving patients with a permanent colostomy or ileostomy [25].

The current study also analysed the risk factors associated with overall and major postoperative morbidity. In particular, severe intraperitoneal adhesion was significantly associated with major morbidity, which has also been identified in various previous studies [34, 35]. RT delivered to the pelvic/abdominal region could contribute to the formation of adhesions and fibrosis, perhaps as a result of vascular damage, which could cause stenosis, fistula and even death [35]. Furthermore, adhesions make subsequently intraperitoneal operations more difficult, and put the patient at higher risk for complications, such as enterotomy, fistula or injury to other intraperitoneal organs.

In multivariate analysis, preoperative anaemia was found to contribute significantly to overall postoperative morbidity, while ASA grades III–V, severe intraperitoneal adhesion and RTOG/EORTC grade V morbidity were significantly associated with major morbidity. Other predisposing factors associated with postoperative morbidity include diabetes mellitus, smoking, hypertension, previous abdominal surgery, concurrent chemotherapy and cumulative dosage, which have been reported in previously studies; however, we did not identify a significant correlation between these parameters and morbidity, probably because the limited number of patients enrolled in our study.

The present study had a number of limitations beyond its retrospective bias. It involved a single-institution sample at a tertiary-care referral centre. The true prevalence of CRE requiring surgery in cervical cancer patients remains unknown because of the paucity of prospective population studies. In addition, patients in the RRT group tended to be older, in worse condition and necessitated a far higher total radiation dose, which could be a confounding factor. However, the key objective of our study was to analyse the characteristics of CRE patients after they had received two treatment modalities (RRT vs RS + RT) for cervical cancer. The study was not novel but is of clinical importance and interest for clinicians regarding the evaluation of the disease course and prognosis.

## Conclusions

Radiation-induced late morbidity tended to be more severe in the RRT group, with more patients suffering from RTOG/EORTC grade IV morbidity. Although there were no significant differences in postoperative morbidity, mortality and rate of reoperation, more patients in the RRT group will inevitably suffer from a permanent stoma. Aggressive resection was feasible in dealing with late morbidities induced by pelvic RT and could be adopted with acceptable levels of surgical complications and postoperative mortality. Severe intra-abdominal adhesion, ASA grades III–V and RTOG grade IV morbidity were found to be predicators of major morbidity.

## Abbreviations

CRE: Chronic Radiation Enteropathy
RRT: Radical Radiotherapy
RS + RT: Radical Surgery plus Radiotherapy
RTOG/EORTC: the Radiation Therapy Oncology Group and the European Organization for Research and Treatment of Cancer
SCRP: Severe Chronic Radiation Proctitis
ASA: American Society of Anesthesiologists
MODS: Multiple organ dysfunction syndrome
